# Audiological and Other Factors Predicting the Presence of Misophonia Symptoms Among a Clinical Population Seeking Help for Tinnitus and/or Hyperacusis

**DOI:** 10.3389/fnins.2022.900065

**Published:** 2022-07-05

**Authors:** Hashir Aazh, Mercede Erfanian, Ali A. Danesh, Brian C. J. Moore

**Affiliations:** ^1^Audiology Department, Royal Surrey NHS Foundation Trust, Guildford, United Kingdom; ^2^Department of Communication Sciences & Disorders, Florida Atlantic University, Boca Raton, FL, United States; ^3^Faculty of Engineering and Physical Sciences (FEPS), University of Surrey, Guildford, United Kingdom; ^4^UCL Institute for Environmental Design and Engineering, The Bartlett, University College London, London, United Kingdom; ^5^Cambridge Hearing Group, Department of Psychology, University of Cambridge, Cambridge, United Kingdom

**Keywords:** misophonia, hyperacusis, hearing loss, tinnitus, uncomfortable loudness levels

## Abstract

This paper evaluates the proportion and the audiological and other characteristics of patients with symptoms of misophonia among a population seeking help for tinnitus and/or hyperacusis at an audiology clinic (*n* = 257). To assess such symptoms, patients were asked “over the last 2 weeks, how often have you been bothered by any of the following problems? Feeling angry or anxious when hearing certain sounds related to eating noises, lip-smacking, sniffling, breathing, clicking sounds, tapping?”. The results of routine audiological tests and self-report questionnaires were gathered retrospectively from the records of the patients. Measures included: pure tone audiometry, uncomfortable loudness levels (ULLs), and responses to the tinnitus impact questionnaire (TIQ), the hyperacusis impact questionnaire (HIQ), and the screening for anxiety and depression in tinnitus (SAD-T) questionnaire. The mean age of the patients was 53 years (SD = 16) (age range 17 to 97 years). Fifty four percent were female. Twenty-three percent of patients were classified as having misophonia. The presence and frequency of reporting misophonia symptoms were not related to audiometric thresholds, except that a steeply sloping audiogram reduced the likelihood of frequent misophonia symptoms. Those with more frequent misophonia symptoms had lower values of ULLmin (the across-frequency average of ULLs for the ear with lower average ULLs) than those with less frequent or no reported symptoms. The reported frequency of experiencing misophonia symptoms increased with increasing impact of tinnitus (TIQ score ≥9), increasing impact of hyperacusis (HIQ score >11), and symptoms of anxiety and depression (SAD-T score ≥4). It is concluded that, when assessing individuals with tinnitus and hyperacusis, it is important to screen for misophonia, particularly when ULLmin is abnormally low or the TIQ, HIQ or SAD-T score is high. This will help clinicians to distinguish patients with misophonia, guiding the choice of therapeutic strategies.

## Introduction

Tinnitus is the perception of sound without an acoustical source external to the body. Hyperacusis is intolerance of certain everyday sounds, which are perceived as too loud or uncomfortable and cause significant distress and impairment in the individual’s day-to-day activities (Aazh et al., 2016, 2022a). Misophonia is characterized by a decreased tolerance for specific sounds (Jastreboff and Jastreboff, 2002; Brout et al., 2018; Swedo et al., 2022). These sounds are known as “triggers,” and they are usually man or animal-made sounds, and often orofacial sounds (generated by the mouth and nose), such as sniffing and chewing. In addition, there is evidence to suggest that, regardless of the source of the triggers, they share similar properties, including repetition (Brout et al., 2018; Erfanian et al., 2019; Enzler et al., 2021b; Hansen et al., 2021). People with misophonia may also be intolerant of certain visual and tactile stimuli (Kumar et al., 2017, 2021; Rouw and Erfanian, 2018; Schroder et al., 2019; Eijsker et al., 2021b). It may be the case that the action of the trigger-producing person is what causes the reaction, rather than the sound itself (Kumar et al., 2021).

The reported prevalence of misophonia varies from 6 to 19%, although a prevalence as high as 37% has been found (Wu et al., 2014; Zhou et al., 2017; Naylor et al., 2021). The prevalence depends on the population studied and on the way that misophonia is diagnosed; the prevalence differs markedly across populations with and without co-morbid disorders. A growing body of literature shows co-morbidity of misophonia with a range of affective disorders as diagnosed in mental health settings, such as major depressive disorder (MDD), obsessive-compulsive personality disorder (OCPD), and post-traumatic stress disorder (PTSD) (Schroder et al., 2013; Rouw and Erfanian, 2018; Erfanian et al., 2019; Jager et al., 2020b), and developmental disorders like attention-deficit hyperactivity disorder (ADHD) and autism spectrum disorder (ASD) (Jager et al., 2020a; McKay and Acevedo, 2020; Haq et al., 2021). The overlapping symptomology of misophonia and psychiatric, developmental, and audiological disorders makes the diagnosis and treatment complicated.

Although auditory disorders, including tinnitus and hyperacusis, often co-occur with misophonia (Jastreboff and Jastreboff, 2014; Danesh and Aazh, 2020), studies focused on misophonia in the field of audiology are scarce (Porcaro et al., 2019). Nevertheless, audiologists play a key role in providing therapy and support for this patient population. Often, audiologists who are specialized in the management of tinnitus and hyperacusis also provide counseling and sound therapy (Jastreboff and Jastreboff, 2014) and/or audiologist-delivered cognitive behavioral therapy for the management of misophonia (Aazh et al., 2014, 2019b). Although the term misophonia was suggested based on studies related to therapy for tinnitus and hyperacusis (Jastreboff and Jastreboff, 2002), most of the research literature on misophonia comes from the fields of psychiatry, psychology and neuroscience, with little or no attention paid to the audiological profile of the population studied. A possible reason for this is that in the field of audiology misophonia is often considered as a subtype of hyperacusis rather than a distinct disorder (Tyler et al., 2014). Therefore, most research studies in the field of audiology have not distinguished misophonia from hyperacusis (Fackrell et al., 2015; Sheldrake et al., 2015; Zaugg et al., 2016; Aazh et al., 2017).

Most studies of misophonia performed in mental health settings have not conducted full audiological evaluations, but some have performed pure tone audiometry on a sub-group of patients (Sztuka et al., 2010; Schroder et al., 2013, 2014; Jager et al., 2020a,b; Siepsiak et al., 2022). Generally, no hearing loss was found, although some cases of tinnitus and/or hyperacusis were reported. However, Enzler et al. (2021b) conducted a study on the development of a psychoacoustic test for assessment of misophonia and reported that among 78 patients with misophonia diagnosed via the MisoQuest questionnaire (Siepsiak et al., 2020), 17 reported hearing problems, 14 had tinnitus, and 55 had hyperacusis. These results suggest that hearing loss, tinnitus and hyperacusis may not be uncommon among patients with misophonia.

Published studies have not assessed the relationship between hearing-related variables and misophonia. In theory, hearing loss could affect the experience of misophonia. The trigger sounds for misophonia often have a spectrum that is dominated by high-frequency components (Dacremont, 1995; Enzler et al., 2021b). A steeply sloping audiogram, with the greatest loss at high frequencies, would reduce the audibility of such sounds, perhaps making it less likely for an individual to have misophonia or reducing the severity of misophonia. On the other hand, people with hearing loss also often experience loudness recruitment, a more rapid than normal growth of loudness with increasing sound level once the sound becomes audible (Moore and Glasberg, 2004). Hence a sound that is only just above the detection threshold may be of moderate loudness and may be annoying. Analysis of the audiometric characteristics of people with misophonia can indicate if hearing loss influences the likelihood or severity of misophonia.

An audiological measure that is often used in the assessment and diagnosis of hyperacusis is the uncomfortable loudness level (ULL) (Aazh and Moore, 2017b). People with hyperacusis often have lower ULLs than people without hyperacusis (Blaesing and Kroener-Herwig, 2012; Formby et al., 2015). In addition, the difference between ULLs at 1 and 8 kHz, a measure of the variation of ULLs across frequency, may be an indicator of a dislike of specific sounds, especially high-frequency sounds. Aazh and Moore (2017b) reported that among patients seeking help for tinnitus and/or hyperacusis the difference between ULLs at 1 and 8 kHz was ≥20 dB for about 10%, perhaps indicating misophonia. Siepsiak et al. (2022) compared ULLs for 62 patients with misophonia and 51 individuals with no sound sensitivity symptoms. The average ULL across ears was about 85 dB HL (standard deviation, SD = 16 dB) for the misophonia group and 90 dB HL (SD = 14 dB) for the control group, but the difference was not statistically significant, perhaps because of the large SD within each group.

Another audiological factor that may be relevant to misophonia is asymmetrical hearing threshold levels (HTLs) or ULLs (i.e., between-ear differences). A large between-ear difference in ULLs might indicate some specific abnormality in monaural pathways. For example, a disorder of the olivo-cochlear efferent system, which reduces the gain of the cochlea in response to high-level sounds, might increase sound sensitivity (Guinan, 2018), and the effect might differ across ears depending on where in the auditory system the disorder originates. On the other hand, if a global psychological or neurological component is predominant in producing hyperacusis and misophonia, then it seems unlikely that it would affect one ear more than the other.

Although to our knowledge ear asymmetry in HTLs or ULLs has not been investigated among patients with misophonia, some reports suggest the presence of asymmetrical HTLs and ULLs among patients with severe hyperacusis (Aazh and Moore, 2017b,^2018^). This is relevant to misophonia as Jastreboff and Jastreboff (2015) reported that misophonia is almost always present in cases of severe hyperacusis. Aazh and Moore (2018) reported that 6 out of 13 patients with severe hyperacusis had an interaural asymmetry between 5 and 12 dB in average ULLs and 5/13 had an interaural asymmetry between 5 and 16 dB in average HTLs. However, due to the small sample size they were not able to assess if greater interaural asymmetry was related to the severity of hyperacusis.

Finally, it is not clear if the likelihood of a person experiencing misophonia is related to whether or not they suffer from distressing tinnitus and/or hyperacusis. Distressing tinnitus and/or hyperacusis may increase anxiety and depression (Aazh and Moore, 2017a), making the individual more likely to develop a strong reaction to trigger sounds, i.e., misophonia. Alternatively, tinnitus may distract the individual, preventing them from attending to potentially annoying trigger sounds. Past studies have not assessed the relationship between the impact of tinnitus and/or hyperacusis and symptoms of misophonia.

The first aim of the current study was to assess the proportion of patients with symptoms of misophonia among a clinical population of patients seeking help for tinnitus and hyperacusis. We predicted that this proportion would be higher than for the general population (Wu et al., 2014; Zhou et al., 2017; Rouw and Erfanian, 2018).

The second aim was to compare the audiological characteristics and severity of tinnitus, hyperacusis, anxiety and depression among patients who reported different frequencies of experiencing symptoms of misophonia in a two-week period (i.e., 0-1 days, 2-6 days, 7-10 days, and 11-14 days). We predicted that a sloping audiogram, with greater hearing loss at high frequencies would be associated with a smaller number of days of experiencing misophonia symptoms and that lower ULLs and more severe tinnitus, hyperacusis, anxiety and depression would be associated with a greater frequency of experiencing of misophonia symptoms.

The results were intended to inform those working in audiology clinics of the likelihood of misophonia among their patients and of factors that are related to it, i.e., factors that increase the probability of misophonia being present. This information could be used to guide the choice of therapy.

## Materials and Methods

### Ethical Approval

The study was registered and approved as a clinical audit by the Quality Governance Department at the Royal Surrey NHS Foundation Trust (RSFT). The need for patient consent was waived as this was a retrospective analysis of available clinical data. Analysis of the data was approved by the South West-Cornwall and Plymouth Research Ethics Committee and the Research and Development department at the RSFT (Project ID: 182924).

### Study Design and Patients

This was a retrospective cross-sectional study conducted at the Tinnitus and Hyperacusis Therapy Specialist Clinic (THTSC), RSFT, Guildford, United Kingdom. Data were included for all patients who attended the THTSC seeking help for tinnitus and/or hyperacusis in 2019-2020 and who answered a question assessing symptoms of misophonia (*n* = 257). Administration of the self-report questionnaires (including the question about misophonia) and audiological measurements included in this study were part of the routine care for patients at THTSC. This routine care did not include the administration of validated questionnaires for assessing misophonia; this issue is addressed in the Discussion section.

Demographic data for the patients, results of their audiological investigations and the outcomes of their self-report questionnaires were imported from their records held at the Audiology Department. All questionnaires were completed prior to the start of any treatment, at each patient’s first visit to the clinic. Patients completed the questionnaires in the clinic waiting area without involvement of their audiologist. The mean age of the patients was 53 years (SD = 16 years) (age range = 17 - 97 years). Fifty four percent (139/257) were female.

### Audiological Measures

Audiological measures were:

(1) Pure tone audiogram measured using the procedure recommended by the British Society of Audiology (BSA, 2011a), but with some modifications proposed by Aazh and Moore (2017c) to limit discomfort. The starting presentation level at 0.25, 0.5, 2, 3, 4, 6, and 8 kHz was equal to the HTL at the adjacent frequency (e.g., if the HTL at 1 kHz was 20 dB HL, the starting level for measuring the HTL at 2 kHz was 20 dB HL, instead of 50 dB HL as recommended by the BSA). The severity of hearing loss was categorized based on the values of the PTA across the frequencies 0.25, 0.5, 1, 2, and 4 kHz, as recommended by the British Society of Audiology (BSA, 2011a): Mild (20 – 40 dB HL), Moderate (41 – 70 dB HL), Severe (71 – 95 dB HL) and Profound (over 95 dB HL). To explore asymmetries in HTLs across the ears, patients were classified into five groups based on the between-ear difference in PTA: <5 dB, ≥5 and <10 dB, ≥10 and <20, ≥20 and <30, and ≥30. The absolute values of the differences in HTLs between 8 and 1 kHz, referred to here as HTL slope were calculated separately for the right and left ears.
(2) ULLs measured following the BSA recommended procedure (BSA, 2011b), but with the modifications proposed by Aazh and Moore (2017c), to limit discomfort. The instructions were “I will gradually make the sound louder in your ear, and you must press the button (or raise your hand) *as soon as* the sound becomes uncomfortable (uncomfortably loud). This is not a test to find the loudest sound you can tolerate; it is a test to find what level of sound you find uncomfortable. You should press the button (or raise your hand) only when the sound becomes uncomfortable; but make sure you press (raise) it as soon as the sound reaches that level.” The starting presentation level was equal to the measured HTL at the test frequency. In addition, levels above 80 dB HL were not used. If the ULL was not reached at 80 dB HL, the ULL at the test frequency was recorded as 85 dB HL. The across-frequency average (0.25, 0.5, 1, 2, 3, 4, 6, and 8 kHz) ULL for the ear with lower average ULL is denoted ULLmin. When ULLmin was ≤ 77 dB HL, hyperacusis was deemed to be present (Aazh and Moore, 2017b). Patients were diagnosed with severe hyperacusis if the ULL for any frequency for either ear was 30 dB HL or less (Aazh and Moore, 2018). To explore asymmetries in ULLs across the ears, patients were classified into three groups based on the between-ears difference in average ULLs (across 0.25, 0.5, 1, 2, 3, 4, 6, and 8 kHz): symmetrical (between-ear difference <5 dB), mildly asymmetrical (between-ear difference ≥5 dB and <10 dB), asymmetrical (between-ear difference ≥ 10 dB). The absolute values of the differences in ULLs between 8 and 1 kHz (i.e., ULL at 8 kHz minus ULL at 1 kHz), referred to here as ULL slope, were calculated separately for the right and left ears.

### Questionnaires

#### Assessment of Misophonia Symptoms

Item 4 of the Sound Sensitivity Symptoms Questionnaire (SSSQ) (Aazh et al., 2022a) was used to identify patients with symptoms of misophonia. This item asks, “Over the last 2 weeks, how often have you been feeling angry or anxious when hearing certain sounds related to eating noises, lip smacking, sniffling, breathing, clicking sounds, tapping?” The response choices are: 0-1 days, 2-6 days, 7-10 days and 11-14 days. Scores of 0 were assigned for 0-1 days, 1 for 2-6 days, 2 for 7-10 days and 3 for 11-14 days. Patients who scored 2 or 3 on this item (i.e., reporting feeling anxious or angry more than half of the days) were classified as having frequent symptoms of misophonia. This is denoted Miso Cat 1. Patients with scores of 0 or 1 (i.e., reporting feeling anxious or angry less than half of the days) were classified as having no or less frequent misophonia symptoms. This is denoted Miso Cat 0. In some of the analyses that follow, the actual score for item 4 of the SSSQ was used. This is denoted SSSQ4.

#### Screening for Anxiety and Depression in Tinnitus

The screening for anxiety and depression (SAD-T) questionnaire contains four items that match those for the physical health questionnaire (PHQ-4; Kroenke et al., 2009). Each item is rated on a four-point Likert scale. Two items relate to experiences of anxiety and worry and two relate to the experience of anhedonia and feeling down, depressed or hopeless. The response choices are: 0-1 days, 2-6 days, 7-10 days and 11-14 days. Scores of 0 were assigned for 0-1 days, 1 for 2-6 days, 2 for 7-10 days and 3 for 11-14 days. Cronbach’s alpha for the SAD-T, based on responses from a tinnitus and hyperacusis clinical population, is 0.91 (Aazh et al., 2022a). The overall score for the SAD-T ranges from 0 to 12. Scores of 4 or more indicate symptoms of anxiety and/or depression. This was calculated but not reported during a study on the acceptability and relevance of psychological questionnaires in the assessment of patients with tinnitus and/or hyperacusis (Aazh and Moore, 2017d).

#### Questions About History of Mental Health

Given the high prevalence of mental illness among patients seeking help for tinnitus and/or hyperacusis, the patients were asked several questions about mental health as part of routine history taking (Aazh and Moore, 2017d; Aazh et al., 2018). The questions were: (1) Do you have any history of mental illness? (2) Have you seen mental health professionals? (3) While you were growing up during the first 18 years of life did your parent(s) have depression or mental illness? The responses for these questions were “yes” or “no.” The third question is taken from the questionnaire for Adverse Childhood Experiences (Felitti et al., 1998).

#### Hyperacusis Impact Questionnaire

The hyperacusis impact questionnaire (HIQ) has eight items assessing the impact of hyperacusis on the patient’s life. The HIQ asks respondents how often (in number of days in the last 14 days) each of several situations occurred because of certain environmental sounds that seemed too loud to them, but that other people could tolerate well. Reponses choices and the score for each choice were the same as for the SAD-T, as described above. Cronbach’s alpha for the HIQ is 0.93. The overall score ranges from 0 to 24. Scores above 11 indicate a clinically significant impact of hyperacusis (Aazh et al., 2022a).

#### Tinnitus Impact Questionnaire

This 7-item questionnaire assesses how often respondents experience a number of problems because of hearing a sound in their ears or head with no external source (e.g., buzzing, high-pitched whistle, hissing), over a two-week period. Reponses choices and the score for each choice were the same as for the SAD-T, as described above. Cronbach’s alpha for the TIQ is 0.89 (Aazh et al., 2022b). The overall score ranges from 0 to 21. A score below 5 indicates no impact of tinnitus, a score of 5 or 6 indicates mild impact, a score of 7 or 8 indicates moderate impact, and a score of 9 or more indicates a severe impact (Aazh et al., 2022b).

### Data Analyses

The data were anonymized prior to statistical analysis. Descriptive statistics for the demographic variables, hearing thresholds and ULLs, and the scores for the self-report questionnaires were calculated.

Welch’s *t*-tests (Delacre et al., 2017) and chi-squared (χ^2^) tests were used to compare audiological variables across frequencies and to assess the differences in the scores for the questionnaires between Miso Cat 1 and Miso Cat 0. Cohen’s *d* was calculated to assess effect sizes (ES) based on mean comparison for unequal variances (Lakens, 2013; Delacre et al., 2017).

One-way analyses of variance (ANOVA) were used to assess the differences in scores for the HIQ, TIQ, SAD-T, ULLmin and PTA across ears among patients with scores of 0, 1, 2, and 3 for item 4 of the SSSQ (SSSQ4 score). The Šídák method was used for post hoc tests (Kirk, 2012). ES values following ANOVA were assessed using the ξ^2^ measure (Smithson, 2001).

Spearman correlation was used to assess the relationships between SSSQ4 scores and scores for the HIQ, TIQ, SAD-T, ULLmin, PTA across ears, HTL and ULL slopes and age. The strength of the correlation coefficient (ρ) was considered as weak if ρ < 0.2, moderate if ρ was between 0.2 and 0.5, and strong if ρ > 0.5 (Cohen, 1988; Hemphill, 2003). Variables that were significantly correlated with SSSQ4 scores were included in a logistic regression model to assess whether the SSSQ4 score (dependent variable) was related to ULLmin, scores for the HIQ, TIQ, SAD-T, and ULL and HTL slopes (independent variables). Odds ratios (ORs) and their 95% confidence intervals were obtained, both unadjusted and adjusted for (a) age and gender (b) categories of tinnitus impact as measured via the TIQ, (c) hyperacusis impact as measured via the HIQ, (d) anxiety and depression as measured via the SAD-T, (e) hyperacusis as measured via ULLmin, (f) ULL slope, and (g) HTL slope. Hearing loss categories and between-ear differences in ULLs and PTA were not included in the model as they were not correlated with SSSQ4 scores. The *p* value required for statistical significance was *p* < 0.05.

The analyses were restricted to patients with complete data for all variables required for a particular analysis. The number of patients included in each analysis (*n*) is reported. The STATA program (version 13) (StataCorp, 2013) and MATLAB 2020a (The MathWorks, 2020) were used for statistical analyses.

## Results

### Characteristics of the Study Population

The means and SDs of the HTLs and ULLs for each ear and each frequency are shown in Table 1. The grand mean PTA across ears was 22 dB HL (SD = 15 dB) (*n* = 244). The grand mean PTA for the better ear was 18 dB HL (SD = 13 dB). The grand mean PTA for the worse ear was 26 dB HL (SD = 19 dB). Based on the PTA for the better ear, 65% of the patients had no hearing loss, 28% had mild hearing loss, and 7% had moderate hearing loss. Based on the PTA for the worse ear, 49% of the patients had no hearing loss, 34% had mild hearing loss, 13.5% had moderate hearing loss, 2.9% had severe hearing loss and 0.8% had profound hearing loss.

**TABLE 1:** Means (SDs) of hearing threshold levels (HTLs) and uncomfortable loudness levels (ULLs) in dB HL for each ear of the study population across different frequencies.

	Frequency, kHz							
	0.25	0.5	1	2	3	4	6	8
HTL right	18 (15) *n* = 247	18 (17) *n* = 247	19 (18) *n* = 247	21 (19) *n* = 247	26 (20) *n* = 218	30 (22) *n* = 247	38 (25) *n* = 218	36 (28) *n* = 247
HTL left	18 (16) *n* = 246	19 (17) *n* = 248	19 (18) *n* = 248	23 (20) *n* = 248	29 (22) *n* = 222	34 (23) *n* = 247	39 (25) *n* = 222	40 (28) *n* = 247
ULL right	78 (10) *n* = 196	78 (9) *n* = 198	79 (8) *n* = 198	79 (9) *n* = 198	79 (9) *n* = 170	79 (9) *n* = 196	79 (10) *n* = 165	77 (12) *n* = 179
ULL left	78 (10) *n* = 195	79 (9) *n* = 198	80 (9) *n* = 196	79 (9) *n* = 195	80 (8) *n* = 167	80 (8) *n* = 189	79 (9) *n* = 163	77 (12) *n* = 182

*The number of patients included in each analysis is indicated by n.*

For 64% of the patients (156/244), there was less than a 5-dB difference in PTA between the two ears. The difference in PTA between ears was ≥ 5 and < 10 dB for 20% of cases, ≥ 10 and < 20 dB for 5% of cases, ≥ 20 and < 30 dB for 4.5% of cases, and ≥ 30 dB for 6.2% of cases. The mean HTL slope was 22.7 dB (SD = 19.5 dB) for the left ears and 20 dB (SD = 19 dB) for the right ears. The HTL slope was ≥ 20 dB, for at least one ear, for 58% of the patients (143/248).

The grand average ULL across 0.25, 0.5, 1, 2, 4, 6, and 8 kHz and across ears was 78.5 dB HL (SD = 8.3) (*n* = 169). The average value of ULLmin was 77.7 dB HL (SD = 9) (*n* = 191). ULLmin values were 77 dB HL or below, suggesting hyperacusis for 30% of patients. About 1.5% of the patients were diagnosed with severe hyperacusis, based on them having a ULL of 30 dB HL or less for any frequency for either ear (Aazh and Moore, 2018). ULLs were symmetrical for 83% of patients, mildly asymmetrical for 14% and asymmetrical for 2.4%. The mean ULL slope was 5 dB (SD = 8 dB) for both ears. About 11.5% of patients had a ULL slope ≥ 20 dB for at least one ear.

For the study population, the mean scores for the HIQ, TIQ and SAD-T were 8 (SD = 7.5, *n* = 224), 8.4 (SD = 6, *n* = 170), and 4 (SD = 4, *n* = 253), respectively. Based on scores for the HIQ, 30% of patients had hyperacusis. Based on scores for the TIQ, 28% of patients had no tinnitus handicap, 20.5% had a mild tinnitus handicap, 10.5% had a moderate tinnitus handicap, and 41% (70/170) had a severe tinnitus handicap. Based on scores for the SAD-T, 44.5% of patients had symptoms of anxiety and/or depression. About 47% of the patients (113/241) reported a history of mental illness, 39% (94/240) reported seeing mental health professionals, and 31.5% reported that when they were under 18 years of age at least one of their parents had mental illness.

### Comparison of Miso Cat 0 and Miso Cat 1

**FIGURE 1:**
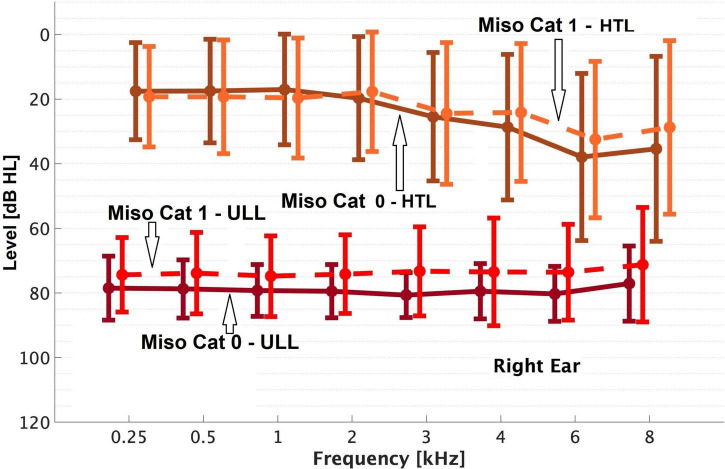
Means and SDs (error bars) of the uncomfortable loudness levels (ULLs) and hearing threshold levels (HTLs) ‘of’ the right ear for Miso Cat 0 and Miso Cat 1 groups.

**FIGURE 2:**
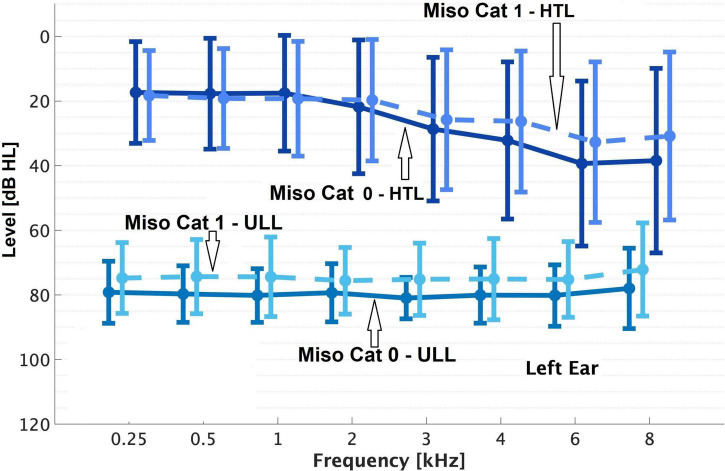
As Figure 1 but for the left ear.

Overall, 23% of patients (59/257) were classified as Miso Cat 1. Patients in Miso Cat 1 were younger on average than those in Miso Cat 0 (Table 2). The percentage of females was 61% for Miso Cat 1 and 52% for Miso Cat 0, and the difference was not significant (χ^2^ = 1.5, *p* = 0.22). There was no significant difference in PTA between those in Miso Cat 0 and those in Miso Cat 1, as shown in Figures 1, 2.

**TABLE 2:** Results of independent-samples Welch’s *t*-tests comparing the PTA (pure tone average) averaged across ears, between-ears difference in PTA, ULLmin (across-frequency average uncomfortable loudness level for the ear with lower average ULL), between-ears difference in average ULL, ULL slope (the value of the difference in ULLs between 8 and 1 kHz) for each ear and averaged across ears, HTL slope (absolute values of the differences in hearing threshold levels between 8 and 1 kHz) for each ear and averaged across ears, scores for the TIQ (Tinnitus Impact Questionnaire), HIQ (Hyperacusis Impact Questionnaire), SAD-T (Screening for Anxiety and Depression-Tinnitus), and age for groups Miso Cat 0 and Miso Cat 1. Significant *p* values are indicated in bold font.

	Miso Cat 0 Mean (SD)	Miso Cat 1 Mean (SD)	Difference: mean and 95% confidence intervals (CI)	*P*-value	ES and 95% CI
PTA across ears	22 (15.5) *n* = 186	22.5 (14) *n* = 58	−0.61 (−4.9 to 3.7)	0.78	−0.04 (−0.33 to 0.25)
Between-ears difference in PTA (dB)	6.8 (11) *n* = 186	9.0 (15.5) *n* = 58	−2.2 (−6.6 to 2.2)	0.31	−0.17 (−0.47 to 0.12)
ULLmin (dB HL)	79 (8) *n* = 145	74 (11) *n* = 46	5.0 (1.5 to 8.5)	**0.006**	0.56 (0.22 to 0.91)
Between-ears difference in average ULL (dB)	1.9 (2.9) *n* = 131	2.7 (3.4) *n* = 38	−0.8 (−2.0 to 0.4)	0.18	−0.27 (−0.63 to 0.095)
ULL slope for right ears (dB)	3.8 (6.5) *n* = 139	9 (10.8) *n* = 40	−5.1 (−8.7 to −1.6)	**0.006**	−0.67 (−1.04 to −0.29)
ULL slope for left ears (dB)	4.1 (6.7) *n* = 138	9.0 (10.4) *n* = 44	−4.8 (−8.2 to −1.5)	**0.005**	−0.62 (−0.98 to −0.26)
ULL slope averaged across ears (dB)	4.2 (6.1) *n* = 132	8.7 (9.4) *n* = 38	−4.5 (−7.8 to −1.3)	**0.007**	−0.64 (−1.02 to −0.26)
HTL slope for right ears (dB)	21.7 (20) *n* = 189	15.4 (16) *n* = 58	6.2 (1.1 to 11.3)	**0.017**	0.33 (0.03 to 0.63)
HTL slope for left ears (dB)	24.2 (20) *n* = 189	18.1 (15) *n* = 58	6.1 (1.1 to 11.0)	**0.016**	0.31 (0.017 to 0.61)
HTL slope averaged across ears (dB)	23.0 (18) *n* = 188	16.8 (14) *n* = 58	6.2 (1.8 to 10.6)	**0.007**	0.36 (0.06 to 0.65)
TIQ score (0-21)	6.8 (4.9) *n* = 131	13.7 (6.6) *n* = 39	−7.0 (−9.2 to −4.7)	** < 0.0001**	−1.3 (−1.7 to −0.86)
HIQ score (0-24)	5.7 (5.9) *n* = 173	16.0 (6.8) *n* = 51	−10.3 (−12.4 to −8.2)	** < 0.0001**	−1.7 (−2.1 to −1.28)
SAD-T score (0-12)	3 (3.4) *n* = 195	7.5 (4.1) *n* = 58	−4.5 (−5.7 to −3.3)	** < 0.0001**	−1.3 (−1.6 to −0.92)
Age (years)	54.5 (17) *n* = 198	49.5 (12) *n* = 59	5.0 (1.0 to 8.9)	**0.014**	0.31 (0.016 to 0.6)

*The sixth column shows ES values based on Cohen’s d with 95% CIs.*

Based on the PTA for the better ear, among the 58 patients in Miso Cat 1, 59% had no hearing loss, 38% had mild hearing loss, and 3.5% had moderate hearing loss. Among the 190 patients in Miso Cat 0, 67% had no hearing loss, 24% had mild hearing loss, and 8% had moderate hearing loss. Based on the PTA for the worse ear, among the patients in Miso Cat 1, 45% had no hearing loss, 38% had mild hearing loss, 14% had moderate hearing loss, and 3.5% had severe hearing loss. Among the patients in Miso Cat 0, 50% had no hearing loss, 33% had mild hearing loss, 13% had moderate hearing loss, 3% had severe hearing loss, and 1% had profound hearing loss. The differences in distributions of hearing loss categories for the better and worse ears between Miso Cat 1 and Miso Cat 0 were not statistically significant (χ^2^ = 5.08, *p* = 0.079 and χ^2^ = 1.3, *p* = 0.86, respectively).

The HTL slope averaged across ears was ≥ 20 dB for 52% of those in Miso Cat 1 and for 59.5% of those in Miso Cat 0 (χ^2^ = 1.1, *p* = 0.3). The PTA differed across ears by less than 5 dB for 65.5% of those in Miso Cat 1 and 63.4% of those in Miso Cat 0. The difference in PTA across ears was ≥5 and <10 dB for 17% of cases in Miso Cat 1 and 21% of Miso Cat 0, ≥10 and <20 dB for 3. 5% of those in Miso Cat 1 and 5.9% of Miso Cat 0, ≥20 and <30 dB for 5.3% of those in Miso Cat 1 and 4.3% of those in Miso Cat 0, and ≥30 dB for 8.6% of those in Miso Cat 1 and 5.4% of those in Miso Cat 0. The proportions of patients falling in each asymmetry category did not differ significantly for Miso Cat 1 and Miso Cat 0 (χ^2^ = 1.7, *p* = 0.79).

As shown in Figures 1, 2, those in Miso Cat 1 had significantly lower (worse) mean ULLs than those in Miso Cat 0 for all frequencies and both ears (all *p* < 0.01). The ULL slope averaged across ears was ≥20 dB for 26% of patients (*n* = 12/46) in Miso Cat 1 compared to 7% (*n* = 10/145) in Miso Cat 0, and this difference in proportions was significant (χ^2^ = 12.6, *p* < 0.001).

Among patients in Miso Cat 1 (*n* = 38), ULLs were symmetrical for 82%, mildly asymmetrical for 16% and asymmetrical for 3%. Corresponding values for those in Miso Cat 0 were 84, 14, and 2%. The proportions of patients falling in each asymmetry category did not differ significantly for Miso Cat 1 and Miso Cat 0 (χ^2^ = 0.12, *p* = 0.94).

Among patients in Miso Cat 1, based on TIQ scores there was no impact of tinnitus for 8% (3/39), a mild impact for 13% (5/39), a moderate impact for 5% (2/39), and a severe impact for 74% (29/39). Among patients in Miso Cat 0, corresponding values were 34% (44/131), 23% (30/131), 12%% (16/131), and 31% (41/131). The proportions falling in the different tinnitus impact categories differed significantly between Miso Cat 1 and 0 (χ^2^ = 23.7, *p* < 0.001), the impact of tinnitus generally being greater for those in Miso Cat 1.

Based on the HIQ score, 73% (37/51) of those in Miso Cat 1 had a significant impact of hyperacusis compared to 18% (31/173) of those in Miso Cat 0, and this difference in proportions was significant (χ^2^ = 55.6, *p* < 0.001).

Based on the SAD-T score, 81% (47/58) of patients in Miso Cat 1 had symptoms of anxiety and depression compared to 33.5% (65/194) of patients in Miso Cat 0, and this difference in proportions was significant (χ^2^ = 40.8, *p* < 0.001).

Seventy one percent (40/56) of patients in Miso Cat 1 reported a history of mental illness compared to 39.5% (73/185) in Miso Cat 0, and this difference in proportions was significant (χ^2^ = 17.6, *p* < 0.0001). Fifty seven percent (32/56) of patients in Miso Cat 1 had seen mental health professionals compared to 34% (62/184) in Miso Cat 0, and this difference in proportions was significant (χ^2^ = 9.9, *p* = 0.002). Forty five percent (25/55) of patients in Miso Cat 1 reported that when they were a child at least one of their parents had mental illness compared to 27% (50/183) for Miso Cat 0, and this difference in proportions was significant (χ^2^ = 6.4, *p* = 0.011). Among patients with a history of parental mental illness in their childhood, 59% (43/73) had an abnormal SAD-T score compared to 39.5% (64/162) of those with no history of parental mental illness, and this difference in proportions was significant (χ^2^ = 7.6, *p* = 0.006).

Table 2 shows the results of Welch’s *t*-tests comparing various measures for those in Miso Cat 0 and Miso Cat 1. The mean ULLmin values were significantly lower and total scores for the SAD-T, TIQ, HIQ were significantly worse for Miso Cat 1 than for Miso Cat 0. The ULL slope was higher for Miso Cat 1 than for Miso Cat 0, but the HTL slope was lower for Miso Cat 1 than for Miso Cat 0. There were no significant differences between Miso Cat 1 and 0 in terms of the between-ear differences in ULL or PTA.

### Audiological and Psychological Factors Related to Misophonia Symptoms

Of 257 patients, SSSQ4 scores of 0, 1, 2, and 3 were obtained for 149 (58%), 49 (19%), 21 (8%) and 38 (15%), respectively. As shown in Table 3, there were significant differences in ULLmin, HIQ, TIQ and SAD-T scores among patients with different SSSQ4 scores. Post hoc pairwise comparisons indicated that ULLmin was significantly lower only for patients who scored 3 compared to 0 for SSSQ4 (*p* = 0.013). The other pairwise comparisons were not significant (*p* > 0.05). HIQ scores were significantly worse for patients whose SSSQ4 scores were 3 vs. 0 (*p* < 0.0001), 2 vs. 0 (*p* = 0.009), 3 vs. 1 (*p* < 0.0001) and 3 vs. 2 (*p* < 0.0001). TIQ scores were significantly worse for patients whose SSSQ4 scores were 3 vs. 0 (*p* < 0.0001), 3 vs. 1 (*p* < 0.0001) and 3 vs. 2 (*p* = 0.005). SAD-T scores were significantly worse for patients whose SSSQ4 scores were 3 vs. 0 (*p* < 0.0001), 2 vs. 0 (*p* = 0.001), 3 vs. 1 (*p* < 0.0001) and 3 vs. 2 (*p* = 0.041). The other pairwise comparisons were not significant (*p* > 0.05).

**TABLE 3:** Means (SD) of ULLmin (across-frequency average uncomfortable loudness level for the ear with lower average ULL), PTA (pure tone average) across ears, and scores for the HIQ (Hyperacusis Impact Questionnaire), TIQ (Tinnitus Impact Questionnaire), and SAD-T (Screening for Anxiety and Depression-Tinnitus) for patients giving each SSSQ4 score, indicating the number of days that they were bothered by certain sounds in the last 2 weeks.

Number of days bothered in the last 14 days	0-1 days	2- 6 days	7-10 days	11-14 days	*F*-value degrees of freedom *p*-value Number (*n*)	ES (95%CI)
ULLmin (dB HL)	80 (8.2) *n* = 105	77 (7.3) *n* = 40	74 (7.8) *n* = 16	74 (13) *n* = 30	4.45 3, 187 **0.005** *n* = 191	0.07 (0.007 to 0.13)
PTA across ears (dB HL)	23 (16) *n* = 137	20 (14) *n* = 49	20 (13) *n* = 21	24 (15) *n* = 37	0.59 3, 240 0.62 *n* = 244	0.007 (0 to 0.03)
HIQ score (0-24)	5.1 (5.8) *n* = 129	7.3 (5.9) *n* = 44	10.2 (6.5) *n* = 15	18.4 (5.4) *n* = 36	50.5 3, 220 ** < 0.0001** *n* = 224	0.41 (0.3 to 0.48)
TIQ score (0-21)	6.0 (4.8) *n* = 94	8.8 (4.6) *n* = 37	9.8 (5.4) *n* = 13	15.7 (6.3) *n* = 26	25.5 3, 166 ** < 0.0001** *n* = 170	0.31 (0.19 to 0.41)
SAD-T score (0-12)	2.7 (3.3) *n* = 146	4.1 (3.4) *n* = 49	5.9 (3.6) *n* = 21	8.5 (4.1) *n* = 37	29.6 3, 249 ** < 0.0001** *n* = 253	0.26 (0.16 to 0.34)

*The right-most column shows the outcomes of one-way ANOVAs with factor SSSQ4 score. The number of patients included in each analysis is indicated by n. Significant p values are indicated in bold font. The seventh column shows ES values based on ξ^2^ with 95% CIs.*

There was no significant difference in the average PTA across ears for patients with different SSSQ4 scores.

As shown in Table 4, there was no significant correlation between the SSSQ4 scores and the average PTA values across ears. There were moderate to strong correlations between SSSQ4 scores and scores for the TIQ, HIQ, and SAD-T. Greater frequency of being bothered by certain sounds was associated with greater impact of tinnitus, greater impact of hyperacusis, and greater incidence of symptoms of anxiety and depression. SSSQ4 scores were moderately correlated with ULL slope values; a higher frequency of being bothered by specific sounds was associated with a greater ULL slope. There was a moderate negative correlation between SSSQ4 scores and ULLmin values and weak negative correlations with HTL slopes and with ages.

**TABLE 4:** Spearman correlations (ρ) and corresponding *p* values between the number of days out of 14 when bothered by certain sounds (based on SSSQ4 score) with: PTA (pure tone average) across ears, TIQ (Tinnitus Impact Questionnaire) scores, HIQ (Hyperacusis Impact Questionnaire) scores, SAD-T (Screening for Anxiety and Depression-Tinnitus) scores, ULL slope (the value of the difference in ULLs between 8 and 1 kHz) for each ear and averaged across ears, ULLmin (across-frequency average uncomfortable loudness level for the ear with lower average ULL), HTL slope (value of the differences in hearing threshold levels between 8 and 1 kHz) for each ear and averaged across ears, and age.

PTA across ears	TIQ score	HIQ score	SAD-T score	ULL slope	ULLmin	HTL slope	Age
ρ = 0.014 *p* = 0.82 *n* = 244	ρ = 0.49 *p* **< 0.0001** *n* = 170	ρ = 0.53 *p* **< 0.0001** *n* = 224	ρ = 0.47 *p* **< 0.0001** *n* = 253	ρ = 0.28 *p* = **0.0002** *n* = 170	ρ = −0.29 *p* **< 0.0001** *n* = 191	ρ = −0.16 *p* = **0.015** *n* = 246	ρ = −0.15 *p* = **0.017** *n* = 257

*Each cell also shows the number of patients (n). Significant p values are indicated in bold font.*

The variables that were significantly correlated with SSSQ4 scores were included in logistic regression models to assess their influence on the OR of an SSSQ4 score of 1, 2, or 3 relative to a score of 0. The predictor variables were: (a) absence versus presence of hyperacusis based on ULLmin values; (b) category of tinnitus impact as measured via the TIQ; (c) no versus significant hyperacusis impact as measured via the HIQ; (d) absence versus presence of anxiety and depression as measured via the SAD-T, (e) ULL slope < 20 dB versus ≥ 20 dB; and (f) HTL slope <20 dB versus ≥20 dB. When all measures were treated as independent (columns 2 and 3 of Table 5), the resulting non-adjusted ORs differed significantly from 1 for all predictors, with the largest effects for tinnitus impact category being moderate or severe, significant impact of hyperacusis, and presence of symptoms of anxiety and depression.

**TABLE 5:** Results of a logistic regression model showing the odds ratio (OR) of the SSSQ4 score (dependent variable) relative to a baseline.

	Non-adjusted OR (95% CI)	*P*-value	Adjusted OR (95% CI) *n* = 120	*P*-value
**Hyperacusis** No (ULLmin > 77 dB HL) Yes (ULLmin ≤ 77 dB HL)	1.0 2.45 (1.3 to 4.6) *n* = 191	**0.005**	1.0 1.1 (0.34 to 3.56)	0.86
**Tinnitus impact category** No impact (TIQ score < 5) Mild (TIQ score 5 or 6) Moderate (TIQ score 7 or 8) Severe (TIQ score ≥ 9)	1.0 2.5 (0.9 to 7.14) 6.1 (1.83 to 20.25) 9.3 (3.8 to 23.1) *n* = 170	0.076 **0.003** < **0.0001**	1.0 3.54 (0.93 to 13.5) 4.39 (0.85 to 22.8) 5.42 (1.46 to 20.17)	0.06 0.08 **0.047**
**Hyperacusis impact category** No impact (HIQ score ≤ 11) Significant impact (HIQ score > 11)	1.0 4.56 (2.48 to 8.4) *n* = 224	** < 0.0001**	1.0 3.9 (1.12 to 13.3)	**0.032**
**Anxiety and depression** No (SAD-T score < 4) Yes (SAD-T score ≥ 4)	1.0 5.4 (3.1 to 9.3) *n* = 252	** < 0.0001**	1.0 2.8 (1.03 to 7.4)	**0.044**
**Across-frequency difference in ULLs** No (across ears ULL slope < 20 dB) Yes (across ears ULL slope ≥ 20 dB)	1.0 2.96 (1.15 to 7.63) *n* = 191	**0.025**	1.0 3.8 (0.67 to 21.98)	0.13
**Across-frequency difference in HTLs** No (across ears HT slope < 20 dB HL) Yes (across ears HT slope ≥ 20 dB HL)	1.0 0.59 (0.36 to 0.99) *n* = 248	**0.046**	1.0 0.31 (0.095 to 0.98)	**0.047**

*Variables included in the model were the presence or absence of hyperacusis based on ULLmin (across-frequency average uncomfortable loudness level for the ear with lower average ULL), tinnitus impact category based on scores for the TIQ (Tinnitus Impact Questionnaire), hyperacusis impact category based on scores for the HIQ (Hyperacusis Impact Questionnaire), presence of anxiety and depression symptoms as measured via the SAD-T (Screening for Anxiety and Depression-Tinnitus), presence or absence of across-frequency difference in ULLs based on the average ULL slope (the values of the difference in ULLs between 8 and 1 kHz) across ears, and presence or absence of across-frequency difference in HTLs based on the HTL slope (values of the differences in hearing threshold levels between 8 and 1 kHz) across ears. Unadjusted and adjusted OR values and their 95% confidence intervals (CIs) are shown. The adjusted OR takes into account the effects of age and gender in addition to the effects of other variables in the model. Significant p values are indicated in bold font.*

Columns 4 and 5 of Table 5 show the outcomes of a model including all six independent variables but adjusted for age and gender and taking into account the effect of each of the six variables on the other variables. The number of patients included in the adjusted model was 120, as complete data for all measures were not available for all patients. For this model, the variables that significantly increased the likelihood of an SSSQ4 score above 0 were: (a) a severe impact of tinnitus; (b) a significant impact of hyperacusis; and (c) having symptoms of anxiety and depression. A difference in HTLs across frequency of 20 dB or more, associated with a high-frequency hearing loss, decreased the likelihood of an SSSQ4 score above 0.

## Discussion

Our results showed that 42% of patients seeking help for tinnitus and/or hyperacusis presented with some symptoms of misophonia. Twenty three percent of patients reported being bothered by certain sounds on 7-14 days in the last 14 days (Miso Cat 1). There was no difference in the prevalence of different degrees of hearing loss among patients in Miso Cat 1 and Miso Cat 0, but a significant proportion of patients in both groups (more than 33%) had some degree of hearing loss, indicating that misophonia is not restricted to those with normal hearing. The percentage of patients with hearing loss among those with misophonia symptoms reported here is higher than reported in previous studies. For example, Enzler et al. (2021b) reported that 22% of individuals with misophonia as measured via the MisoQuest had self-reported hearing issues and Siepsiak et al. (2022) reported hearing loss in 16% of participants with misophonia, as diagnosed using the criteria of Schroder et al. (2013). Most of the patients in those studies were recruited via social media, so their study population was different from that for our study.

Although the presence or absence of hearing loss did not seem to be related to the presence of misophonia symptoms, a steep slope of the audiogram, with greater loss at high frequencies, was associated with a reduced risk of misophonia. This probably occurs because some of the triggers for misophonia are sounds whose spectrum is dominated by high frequencies, such as the sound of crispy foods (Dacremont, 1995). Hearing loss at high frequencies reduces the likelihood that such trigger sounds will be audible.

The presence of symptoms of misophonia was not significantly related to between-ear differences in HTL or ULL. This indicates that the underlying mechanism of misophonia is unlikely to be related to asymmetric pathologies of the peripheral auditory pathway; rather, a more central mechanism is involved. This is consistent with imaging studies reporting altered non-auditory areas in the brain among patients with misophonia compared with healthy controls (Kumar et al., 2017; Lin et al., 2020).

In this paper, one of the criteria for indicating the presence of hyperacusis was a ULLmin value ≤77 dB HL (the other criterion was HIQ score). The use of ULLs for diagnosing hyperacusis has been challenged by several authors; some studies have reported that ULLs averaged across frequency were not significantly correlated with self-report measures of hyperacusis (Khalfa et al., 2002; Meeus et al., 2010). In addition, there are differences in the criteria for diagnosing hyperacusis based on ULLs (Goldstein and Shulman, 1996; Anari et al., 1999; Jastreboff and Jastreboff, 2000). For example, Goldstein and Shulman (1996) suggested that ULLs between 80 and 90 dB HL at two or more frequencies indicate mild hyperacusis, ULLs between 65 and 75 dB HL indicate moderate hyperacusis and ULLs below 60 dB HL indicate severe hyperacusis. Anari et al. (1999) suggested 70 dB HL as the cutoff value indicating significant hyperacusis. Jastreboff and Jastreboff (2000) suggested that “threshold of significant hyperacusis is defined as average LDLs below 100 dB HL” (LDL stands for loudness discomfort level, which is another term for ULL). Sheldrake et al. (2015) reported that if a criterion value of 100 dB HL for ULLs averaged across 0.5, 1, 2, and 4 kHz (denoted ULL_0.5–4_) is used, this results in a positive diagnosis for 90% of those with hyperacusis, but results in a high false positive rate of 60% (and a corresponding specificity of only 40%).

Sherlock and Formby (2005) reported that among individuals with no complaint of hyperacusis the average value of ULL_0.5–4_ was 102 dB HL (SD = 12 dB). They showed that 50% of people with no hyperacusis had average ULL_0.5–4_ values less than 105 dB HL, 25% had ULL_0.5–4_ values less than 94 dB HL, and 5% had ULL_0.5–4_ values less than 80 dB HL. To avoid excessive false positives when diagnosing hyperacusis based on ULL_0.5–4_, the lower 95% bound of the global mean for people without hyperacusis can be used as the cutoff; this is obtained by subtracting from that mean 1.96 times the square root of the variance of the mean, giving a value of 80 dB HL based on the data of Sherlock and Formby (2005).

Aazh and Moore (2017b) took a different approach. As noted earlier, they based their analyses on the average ULL across 0.25, 0.5, 1, 2, 3, 4, 6, and 8 kHz for the ear with lower average ULLs, denoted ULLmin. They chose a cutoff value for ULLmin corresponding to the 95% upper bound of the ULLmin values for people with hyperacusis as diagnosed via the score for Hyperacusis Questionnaire (HQ) (Khalfa et al., 2002); this was obtained by adding 1.96 times the square root of the variance of the global mean ULLmin value to the global mean value, based on the clinical data of patients seen in a tinnitus and hyperacusis service. The resulting value was 77 dB HL. For this criterion, anyone with ULLmin ≤ 77 dB HL is diagnosed as having hyperacusis. Based on the data reported by Aazh and Moore (2017b), sensitivity with this criterion is 53% and specificity is 79%. Thus, the cutoff value of 77 dB HL only rarely results in false positives. The ULLmin criterion of 77 dB has recently been used in a study of Enzler et al. (2021a), who proposed a novel method of assessing hyperacusis using psychoacoustic ratings of natural sounds. Their results showed good consistency of the new psychoacoustic method in diagnosing hyperacusis with the ULLmin criterion. Note, however, that whatever criterion is chosen, diagnosis of hyperacusis based on ULLs is imperfect. That is why, in the present study, HIQ scores were used in addition to ULLs.

The average value of ULLmin was 74 dB HL for those in Miso Cat 1 and 79 dB HL for those in Miso Cat 0, and this difference was significant. Also, there was a moderate negative correlation between SSSQ4 scores and ULLmin values. In other words, those with low ULLmin values were more likely to show symptoms of misophonia. In contrast, Siepsiak et al. (2022) reported no statistically significant difference in average ULLs across ears between patients with and without misophonia, although there was a trend in the same direction as found in the present study. The difference across studies may be a consequence of the different study populations, but may also be related to differences in the method for measuring ULLs. The maximum presentation level used by Siepsiak et al. (2022) ranged from 90 to 120 dB HL depending on the test frequency. In our study, the level was limited to 80 dB HL regardless of frequency in order to avoid discomfort, as recommended by Aazh and Moore (2017c).

One concern is the extent to which the cap of 80 dB HL influenced the values of ULLmin. There were very few cases of patients who were classified as having hyperacusis based on ULLmin who did not press the button indicating the onset of discomfort at a level of 80 dB HL or below, so the artificial value of 85 dB was used only rarely, and then usually only for one or two frequencies. Thus, the cap of 80 dB HL had very little influence on the values of ULLmin among those who were classified as having hyperacusis based on ULLmin. For patients who were not classified as having hyperacusis based on ULLmin, 59 and 70 (out of 133) did not press the button at 80 dB HL or below for the left and right ears, respectively. Therefore, the cap of 80 dB HL would have reduced the mean ULLmin value among patients who were not classified as having hyperacusis based on ULLmin values. The overall effect of the cap was to reduce the difference in average ULLmin values for those diagnosed as having versus not having hyperacusis. It is likely that the differences in ULLmin values between those in Miso Cat 0 and Miso Cat 1 would have been even larger if the cap of 80 dB HL had not been imposed.

The ULL slope was significantly higher (steeper) for patients in Miso Cat 1 than for patients in Miso Cat 0. The ULL slope values were moderately correlated with SSSQ4 scores, indicating that patients with misophonia symptoms are likely to be more bothered by high-frequency sounds than by low-frequency sounds. This is consistent with the finding that sounds with strong concentrations of energy in the range 2.5 to 5.5 kHz are associated with auditory perceptual unpleasantness for normal subjects (Halpern et al., 1986; Kumar et al., 2008). The auditory system is maximally sensitive over this frequency range, in that absolute thresholds are lowest, and for a given sound level loudness is greatest (Moore et al., 1997). This sensitivity may be magnified in patients with misophonia, as has been observed for individuals with noise sensitivity (Kliuchko et al., 2016). High sensitivity to high-frequency sounds has also been reported for cases of severe hyperacusis (Aazh and Moore, 2018) and many of the patients in our sample with higher SSSQ4 scores also had hyperacusis as measured via the HIQ and ULLmin.

The proportion of patients who had seen mental health professionals was significantly higher for Miso Cat 1 than for Miso Cat 0. This is consistent with the finding of Kılıç et al. (2021) that contact with mental health services for any psychological problem was more common among those with misophonia than among those without (48 vs. 29%). The present study showed that SAD-T scores were moderately correlated with SSSQ4 scores. This is consistent with other reports of a relationship between misophonia and mental illness (Guetta et al., 2022; Siepsiak et al., 2022). More in-depth investigation is needed to shed light on the directionality of the association between misophonia and psychiatric disorders/symptoms. Specifically, it would be useful to assess whether the chance of being affected by psychiatric disorders is higher when misophonia already exists (Erfanian et al., 2019).

A new finding of our study was that the proportion of patients with a childhood history of parental mental illness was higher for Miso Cat 1 (45%) than for Miso Cat 0 (27%). This is consistent with reports of a higher impact of tinnitus, hyperacusis-induced anxiety, and depression symptoms among patients who reported that during their first 18 years of life their parent(s) suffered from a mental illness (Aazh et al., 2018, 2019a,c, 2020). Mounting evidence suggests that adverse childhood experiences play a major lifelong role in mental and physical problems (Anda et al., 2006, 2010; Erfanian, 2018). Parental mental illness is an important form of adverse childhood experiences (Anda et al., 2006). Future studies should explore the history of exposure to various childhood adverse experiences, ranging from different forms of abuse (physical, emotional, or sexual), neglect (physical and emotional) and various aspects of household dysfunction (substance abuse in the family, parental mental illness, mother treated violently, imprisoned household member, or parental separation) among patients with misophonia (Felitti et al., 1998; Felitti, 2009). This is important because, if a significant relationship exists, the presence of more severe misophonia symptoms in patients could be an indicator of childhood adverse experiences. Patients with a history of childhood adversities often need more complex and in-depth psychological treatments for their mental health should they develop emotional problems (Pigeon et al., 2009; Kajeepeta et al., 2015).

Scores for the TIQ and HIQ were significantly worse for patients in Miso Cat 1 than for those in Miso Cat 0. Also, TIQ scores were moderately correlated with SSSQ4 scores and HIQ scores were strongly correlated with SSSQ4 scores. The adjusted logistic regression model showed that patients with a severe impact of tinnitus and a significant impact of hyperacusis were more likely to have a higher SSSQ4 score. To the best of our knowledge, these are novel findings. This is consistent with the similarity of the neuropathology of misophonia, hyperacusis and tinnitus, as indicated by altered auditory-limbic system connections (Kumar et al., 2017; Lin et al., 2020), micro-structural alternations of white matter in non-auditory regions (Chen et al., 2020; Eijsker et al., 2021a), and functional connectivity among auditory cortex, cerebellum and the limbic system (Kumar et al., 2017; Cai et al., 2019; Eijsker et al., 2021b). A similar relationship has been reported between tinnitus and hyperacusis: patients with a more severe impact of tinnitus also tend to have more severe symptoms of hyperacusis (Aazh and Moore, 2017d; Cederroth et al., 2020; Aazh et al., 2021).

The adjusted logistic regression model also showed that a score of 4 or more for the SAD-T significantly increased the odds of having a higher SSSQ4 score, consistent with the idea that misophonia is associated with anxiety and depression. Given that misophonia leads to significant emotional distress, interpersonal and social difficulties, disability, and interference with daily life, it is not surprising that it contributes to the development of anxiety and depression. Sufferers may also experience functional impairments, such as difficulty in performing their job and concentration difficulties (Swedo et al., 2022). Finally, the adjusted model showed that a slope of the audiogram of 20 dB or more significantly decreased the odds of having a higher SSSQ4 score, consistent with the idea that reduced audibility of high-frequency sounds decreases the chances of misophonia trigger sounds being audible. There is a gap in our understanding of the function of auditory system in this patient population, and future studies should explore other characteristics of the auditory system among patients with misophonia using psycho-acoustic and electrophysiological measures.

This study was based on a retrospective analysis of the available clinical data for patients seen during the years 2019 and 2020. Therefore, we were limited to the measures that were obtained as a part of routine clinical practice at the THTSC during that time. Misophonia was assessed based on only one question (item 4 of the SSSQ). This is not unusual for clinical services, since misophonia questionnaires have not yet been widely adopted by audiologists in day-to-day clinical practice. However, we recognize that using only one question to assess misophonia is not ideal, although it has been done by other researchers for assessing misophonia, hyperacusis severity, hearing impairment and tinnitus severity (Schecklmann et al., 2014; Greenberg and Carlos, 2018; Cederroth et al., 2020; Jaswal et al., 2021). Also, the validity and reliability of using SSSQ4 to assess the frequency of reported misophonia symptoms have not been evaluated. Therefore, the results of our correlational and regression modeling need to be interpreted with caution. To address this limitation, future studies should use validated measures to assess the relationship between misophonia and measures of the impact of tinnitus and hyperacusis, measures of anxiety and depression, and hearing-related variables. Examples of these measures are MisoQuest (Siepsiak et al., 2020), the Amsterdam Misophonia Scale (Schroder et al., 2013; Naylor et al., 2021), the Misophonia Response Scale (Dibb et al., 2021), the Core Discriminant Sounds of Misophonia (Enzler et al., 2021b), the Duke Misophonia Questionnaire (Rosenthal et al., 2021) and the Misophonia Questionnaire (MQ) (Wu et al., 2014).

Another limitation is that all patients were referred to an audiology clinic for tinnitus and/or hyperacusis management. Therefore, our results are probably not representative of the general population or of patients referred to mental health services.

## Conclusions

Among a population seeking help from an audiology clinic for tinnitus and/or hyperacusis, 23% were classified as having misophonia. The presence and frequency of reported symptoms of misophonia were not related to audiometric thresholds, or to the asymmetry of audiometric thresholds across ears, except that a steeply sloping audiogram reduced the likelihood of more frequently reported misophonia symptoms in a 2-week period. The latter effect may reflect the finding that the sounds that trigger misophonia often contain significant energy at high frequencies, and high-frequency hearing loss reduces the likelihood of such sounds being audible. Those with higher SSSQ4 scores had lower values of ULLmin (the across-frequency average of ULLs for the ear with lower average ULLs) than those with lower SSSQ4 scores. The frequency of reported misophonia symptoms as measured via SSSQ4 increased with increasing impact of tinnitus. Using a logistic regression model adjusted for the effects of age and gender, it was found that a TIQ score ≥9 increased the odds of reporting misophonia symptoms by a factor of 5.4. Using the same adjusted model, it was found that an HIQ score >11 (indicating a significant impact of hyperacusis) increased the odds of reporting misophonia symptoms by a factor of 3.9. Using the same adjusted model, it was found that a SAD-T score ≥4 (indicating symptoms of anxiety and depression) increased the odds of reporting misophonia symptoms by a factor of 2.8. We conclude that, when assessing individuals with tinnitus and hyperacusis, it is important to screen for misophonia, particularly when ULLmin is abnormally low or the TIQ, HIQ or SAD-T score is abnormally high. This will help clinicians to distinguish misophonia from similar disorders, guiding the choice of therapeutic strategies.

## Data Availability Statement

The original contributions presented in this study are included in the article/supplementary material. Further inquiries can be directed to the corresponding author.

## Ethics Statement

The study was registered, reviewed and approved as a clinical audit by the Quality Governance Department at RSFT. The need for patient consent was waived as this was a retrospective analysis of available clinical data. Analysis of the data was approved by the South West-Cornwall and Plymouth Research Ethics Committee and the Research and Development department at the RSFT (Project ID: 182924).

## Author Contributions

HA collected the data. HA, ME, AD, and BM collaborated on analysis of the data, interpretations of the results and preparing the manuscript. All authors contributed to the article and approved the submitted version.

## Conflict of Interest

The authors declare that the research was conducted in the absence of any commercial or financial relationships that could be construed as a potential conflict of interest.

## Publisher’s Note

All claims expressed in this article are solely those of the authors and do not necessarily represent those of their affiliated organizations, or those of the publisher, the editors and the reviewers. Any product that may be evaluated in this article, or claim that may be made by its manufacturer, is not guaranteed or endorsed by the publisher.

## References

[B1] Aazh H. Moore B. C. J. (2017b). Factors related to Uncomfortable Loudness Levels for patients seen in a tinnitus and hyperacusis clinic. *Int. J. Audiol.* 56 793–800. 10.1080/14992027.2017.1335888 28622055

[B2] Aazh H. Moore B. C. J. (2017a). Factors associated with depression in patients with tinnitus and hyperacusis. *Am. J. Audiol.* 26 562–569. 29209701 10.1044/2017_AJA-17-0008

[B3] Aazh H. Moore B. C. J. (2017c). Incidence of discomfort during pure-tone audiometry and measurement of uncomfortable loudness levels among People seeking help for tinnitus and/or hyperacusis. *Am. J. Audiol.* 26 226–232. 28810267 10.1044/2017_AJA-17-0011

[B4] Aazh H. Moore B. C. J. (2017d). Usefulness of self-report questionnaires for psychological assessment of patients with tinnitus and hyperacusis and patients’ views of the questionnaires. *Int. J. Audiol.* 56 489–498. 10.1080/14992027.2017.1298850 28277857

[B5] Aazh H. Moore B. C. J. (2018). Prevalence and characteristics of patients with severe hyperacusis among patients seen in a tinnitus and hyperacusis clinic. *J. Am. Acad. Audiol.* 29 626–633. 10.3766/jaaa.17015 29988010

[B6] Aazh H. Landgrebe M. Danesh A. Moore B. C. J. (2019b). Cognitive behavioral therapy for alleviating the distress caused by tinnitus, hyperacusis and misophonia: current perspectives. *Psychol. Res. Behav. Manage.* 23 991–1002. 10.2147/PRBM.S179138 PMC6817772 31749641

[B7] Aazh H. Danesh A. Moore B. C. J. (2019a). Parental mental health in childhood as a risk factor for anxiety and depression among people seeking help for tinnitus and hyperacusis. *J. Am. Acad. Audiol.* 30 772–780. 10.3766/jaaa.18001 30446035

[B8] Aazh H. Landgrebe M. Danesh A. A. (2019c). Parental mental illness in childhood as a risk factor for suicidal and self-harm ideations in adults seeking help for tinnitus and/or hyperacusis. *Am. J. Audiol.* 28 527–533. 31184510 10.1044/2019_AJA-18-0059

[B9] Aazh H. Danesh A. Moore B. C. J. (2021). Internal consistency and convergent validity of the inventory of hyperacusis symptoms. *Ear Hear.* 42 917–926. 10.1097/aud.0000000000000982 33259445

[B10] Aazh H. Hayes C. Moore B. C. J. Danesh A. A. Vitoratou S. (2022a). Psychometric evaluation of the hyperacusis impact questionnaire (HIQ) and sound sensitivity symptoms questionnaire (SSSQ) using a clinical population of adult patients with tinnitus alone or combined with hyperacusis. *J. Am. Acad. Audiol.* 10.1055/a-1780-4002 [Epub ahead of print]. 35196727 PMC9788912

[B11] Aazh H. Hayes C. Moore B. C. J. Vitoratou S. (2022b). Psychometric evaluation of the tinnitus impact questionnaire using a clinical population of adult patients with tinnitus alone or combined with hyperacusis. *Int. J. Audiol*. (under review). 10.1080/14992027.2022.2101027 35916560

[B12] Aazh H. Lammaing K. Moore B. C. J. (2017). Factors related to tinnitus and hyperacusis handicap in older people. *Int. J. Audiol.* 56 677–684. 10.1080/14992027.2017.1335887 28625091

[B13] Aazh H. Langguth B. Danesh A. A. (2018). Parental separation and parental mental health in childhood and tinnitus and hyperacusis handicap in adulthood. *Int. J. Audiol.* 57 941–946. 30272507 10.1080/14992027.2018.1514470

[B14] Aazh H. McFerran D. Salvi R. Prasher D. Jastreboff M. Jastreboff P. (2014). Insights from the first international conference on hyperacusis: causes, evaluation, diagnosis and treatment. *Noise Health* 16 123–126. 10.4103/1463-1741.132100 24804717

[B15] Aazh H. Moore B. C. J. Lammaing K. Cropley M. (2016). Tinnitus and hyperacusis therapy in a UK national health service audiology department: patients’ evaluations of the effectiveness of treatments. *Int. J. Audiol.* 55 514–522. 10.1080/14992027.2016.1178400 27195947 PMC4950421

[B16] Aazh H. Puri B. K. Moore B. C. J. (2020). Parental separation and parental mental health in childhood and risk of insomnia in adulthood among patients with tinnitus. *J. Am. Acad. Audiol.* 31 217–223. 10.3766/jaaa.19023 31287055

[B17] Anari M. Axelsson A. Eliasson A. Magnusson L. (1999). Hypersensitivity to sound–questionnaire data, audiometry and classification. *Scand. Audiol.* 28 219–230. 10572967 10.1080/010503999424653

[B18] Anda R. F. Felitti V. J. Bremner J. D. Walker J. D. Whitfield C. Perry B. D. (2006). The enduring effects of abuse and related adverse experiences in childhood. A convergence of evidence from neurobiology and epidemiology. *Eur. Arch. Psychiatry Clin. Neurosci.* 256 174–186. 10.1007/s00406-005-0624-4 16311898 PMC3232061

[B19] Anda R. Tietjen G. Schulman E. Felitti V. Croft J. (2010). Adverse childhood experiences and frequent headaches in adults. *Headache* 50 1473–1481. 10.1111/j.1526-4610.2010.01756.x 20958295

[B20] Blaesing L. Kroener-Herwig B. (2012). Self-reported and behavioral sound avoidance in tinnitus and hyperacusis subjects, and association with anxiety ratings. *Int. J. Audiol.* 51 611–617. 22443320 10.3109/14992027.2012.664290

[B21] Brout J. J. Edelstein M. Erfanian M. Mannino M. Miller L. J. Rouw R. (2018). Investigating misophonia: a review of the empirical literature, clinical implications, and a research agenda. *Front. Neurosci.* 12:36. 10.3389/fnins.2018.00036 29467604 PMC5808324

[B22] BSA (2011a). *Pure-Tone Air-Conduction And Bone-Conduction Threshold Audiometry With And Without Masking: Recommended Procedure.* Reading: British Society of Audiology.

[B23] BSA (2011b). *Recommended Procedure: Determination Of Uncomfortable Loudness Levels.* Reading: British Society of Audiology.

[B24] Cai W. W. Li Z. C. Yang Q. T. Zhang T. (2019). Abnormal spontaneous neural activity of the central auditory system changes the functional connectivity in the tinnitus brain: a resting-state functional MRI study. *Front. Neurosci.* 13:1314. 10.3389/fnins.2019.01314 31920484 PMC6932986

[B25] Cederroth C. R. Lugo A. Edvall N. K. Lazar A. Lopez-Escamez J.-A. Bulla J. (2020). Association between hyperacusis and tinnitus. *J. Clin. Med.* 9:2412. 10.3390/jcm9082412 PMC7465629 32731492

[B26] Chen Q. Wang Z. D. Lv H. Zhao P. F. Yang Z. H. Gong S. S. (2020). Reorganization of brain white matter in persistent idiopathic tinnitus patients without hearing loss: evidence from baseline data. *Front. Neurosci.* 14:591. 10.3389/fnins.2020.00591 32612504 PMC7308730

[B27] Cohen J. (1988). *Statistical Power Analysis For The Behavioral Sciences.* Mahwah, NJ: Lawrence Erlbaum Associates, Inc. Publishers.

[B28] Dacremont C. (1995). Spectral composition of eating sounds generated by crispy, crunchy and crackly foods. *J. Texture Stud.* 26 27–43. 10.1111/j.1745-4603.1995.tb00782.x

[B29] Danesh A. Aazh H. (2020). Misophonia: a neurologic, psychologic, and audiologic complex. *Hear. J.* 23 20–23.

[B30] Delacre M. Lakens D. Leys C. (2017). Why psychologists should by default use Welch’s t-test instead of Student’s t-test. *Int. Rev. Soc. Psychol.* 30 92–101.

[B31] Dibb B. Golding S. E. Dozier T. H. (2021). The development and validation of the Misophonia response scale. *J. Psychosom. Res.* 149:110587. 10.1016/j.jpsychores.2021.110587 34390941

[B32] Eijsker N. Schroder A. Smit D. J. A. van Wingen G. Denys D. (2021b). Structural and functional brain abnormalities in misophonia. *Eur. Neuropsychopharmacol.* 52 62–71. 10.1016/j.euroneuro.2021.05.013 34273684

[B33] Eijsker N. Schroder A. Liebrand L. C. Smit D. J. A. van Wingen G. Denys D. (2021a). White matter abnormalities in misophonia. *NeuroImage Clin.* 32:102787. 10.1016/j.nicl.2021.102787 34461433 PMC8405911

[B34] Enzler F. Fournier P. Noreña A. J. (2021a). A psychoacoustic test for diagnosing hyperacusis based on ratings of natural sounds. *Hear. Res.* 400:108124. 10.1016/j.heares.2020.108124 33321385

[B35] Enzler F. Loriot C. Fournier P. Norena A. J. (2021b). A psychoacoustic test for misophonia assessment. *Sci. Rep.* 11:11044. 10.1038/s41598-021-90355-8 34040061 PMC8155015

[B36] Erfanian M. (2018). Childhood trauma: a risk for major depression in patients with psoriasis. *Psychiatry Clin. Psych.* 28 378–385. 10.1080/24750573.2018.1452521

[B37] Erfanian M. Kartsonaki C. Keshavarz A. (2019). Misophonia and comorbid psychiatric symptoms: a preliminary study of clinical findings. *Nordic J. Psychiatry* 73 219–228. 10.1080/08039488.2019.1609086 31066600

[B38] Fackrell K. Fearnley C. Hoare D. J. Sereda M. (2015). Hyperacusis Questionnaire as a tool for measuring hypersensitivity to sound in a tinnitus research population. *BioMed Res. Int.* 2015:290425. 10.1155/2015/290425 26557658 PMC4628763

[B39] Felitti V. J. (2009). Adverse childhood experiences and adult health. *Acad. Pediatr.* 9 131–132. 10.1016/j.acap.2009.03.001 19450768

[B40] Felitti V. J. Anda R. F. Nordenberg D. Williamson D. F. Spitz A. M. Edwards V. (1998). Relationship of childhood abuse and household dysfunction to many of the leading causes of death in adults. The adverse childhood experiences (ACE) Study. *Am. Coll. Prevent. Med.* 14 245–258. 10.1016/s0749-3797(98)00017-8 9635069

[B41] Formby C. Hawley M. L. Sherlock L. P. Gold S. Payne J. Brooks R. (2015). A sound therapy-based intervention to expand the auditory dynamic range for loudness among persons with sensorineural hearing losses: a randomized placebo-controlled clinical trial. *Semin. Hear.* 36 77–110. 10.1055/s-0035-1546958 27516711 PMC4906300

[B42] Goldstein B. Shulman A. (1996). Tinnitus-Hyperacusis and loudness discomfort level test- a preliminary report. *Int. Tinnitus J.* 2 83–89. 10753346

[B43] Greenberg B. Carlos M. (2018). Psychometric properties and factor structure of a new scale to measure hyperacusis: introducing the inventory of hyperacusis symptoms. *Ear Hear.* 39 1025–1034. 10.1097/aud.0000000000000583 29742543

[B44] Guetta R. E. Cassiello-Robbins C. Trumbull J. Anand D. Rosenthal M. Z. (2022). Examining emotional functioning in misophonia: the role of affective instability and difficulties with emotion regulation. *PLoS One* 17:e0263230. 10.1371/journal.pone.0263230 35148347 PMC8836307

[B45] Guinan J. J. Jr. (2018). Olivocochlear efferents: their action, effects, measurement and uses, and the impact of the new conception of cochlear mechanical responses. *Hear. Res.* 362 38–47. 10.1016/j.heares.2017.12.012 29291948 PMC5911200

[B46] Halpern D. L. Blake R. Hillenbrand J. (1986). Psychoacoustics of a chilling sound. *Percept. Psychophys.* 39 77–80. 10.3758/bf03211488 3725541

[B47] Hansen H. A. Leber A. B. Saygin Z. M. (2021). What sound sources trigger misophonia? Not just chewing and breathing. *J. Clin. Psychol.* 77 2609–2625. 10.1002/jclp.23196 34115383

[B48] Haq S. S. Alresheed F. Tu J. C. (2021). Behavioral treatment of problem behavior for an adult with autism spectrum disorder and misophonia. *J. Dev. Phys. Disabil.* 33 1005–1015.

[B49] Hemphill J. F. (2003). Interpreting the magnitudes of correlation coefficients. *Am. Psychol.* 58 78–79. 10.1037/0003-066x.58.1.78 12674822

[B50] Jager I. J. de Koning P. Bost T. Denys D. Vulink N. (2020a). Misophonia: phenomenology, comorbidity and demographics in a large sample. *PLoS One* 15:e0231390. 10.1371/journal.pone.0231390 32294104 PMC7159231

[B51] Jager I. J. Vulink N. C. C. Bergfeld I. O. van Loon A. Denys D. (2020b). Cognitive behavioral therapy for misophonia: a randomized clinical trial. *Depress. Anxiety* 38 708–718. 10.1002/da.23127 33336858 PMC8359510

[B52] Jastreboff M. M. Jastreboff P. J. (2002). Decreased sound tolerance and tinnitus retraining therapy (TRT). *Aust. N. Zeal. J. Audiol.* 24 74–84.

[B53] Jastreboff P. J. Jastreboff M. M. (2000). Tinnitus retraining therapy (TRT) as a method for treatment of tinnitus and hyperacusis patients. *J. Am. Acad. Audiol.* 11 162–177. 10755812

[B54] Jastreboff P. J. Jastreboff M. M. (2014). Treatments for decreased sound tolerance (hyperacusis and misophonia). *Semin. Hear.* 35 105–120. 10.1016/B978-0-444-62630-1.00021-4 25726280

[B55] Jastreboff P. J. Jastreboff M. M. (2015). Decreased sound tolerance: hyperacusis, misophonia, diplacousis, and polyacousis. *Handb. Clin. Neurol.* 129 375–387. 10.1016/b978-0-444-62630-1.00021-4 25726280

[B56] Jaswal S. M. De Bleser A. K. F. Handy T. C. (2021). Misokinesia is a sensitivity to seeing others fidget that is prevalent in the general population. *Sci. Rep.* 11:17204. 10.1038/s41598-021-96430-4 34446737 PMC8390668

[B57] Kajeepeta S. Gelaye B. Jackson C. L. Williams M. A. (2015). Adverse childhood experiences are associated with adult sleep disorders: a systematic review. *Sleep Med.* 16 320–330. 10.1016/j.sleep.2014.12.013 25777485 PMC4635027

[B58] Khalfa S. Dubal S. Veuillet E. Perez-Diaz F. Jouvent R. Collet L. (2002). Psychometric normalization of a hyperacusis questionnaire. *J. Otorhinolaryngol. Relat. Spec.* 64 436–442. 10.1159/000067570 12499770

[B59] Kılıç C. Öz G. Avanoğlu K. B. Aksoy S. (2021). The prevalence and characteristics of misophonia in Ankara, Turkey: population-based study. *BJPsych Open* 7:e144. 10.1192/bjo.2021.978 34353403 PMC8358974

[B60] Kirk R. E. (2012). *Experimental Design: Procedures For The Behavioral Sciences.* Thousand Oaks, CA: Sage Publications.

[B61] Kliuchko M. Heinonen-Guzejev M. Vuust P. Tervaniemi M. Brattico E. (2016). A window into the brain mechanisms associated with noise sensitivity. *Sci. Rep.* 6:39236. 10.1038/srep39236 PMC5157031 27976708

[B62] Kroenke K. Spitzer R. L. Williams J. B. Lowe B. (2009). An ultra-brief screening scale for anxiety and depression: the PHQ-4. *Psychosomatics* 50 613–621. 10.1176/appi.psy.50.6.613 19996233

[B63] Kumar S. Dheerendra P. Erfanian M. Benzaquén E. Sedley W. Gander P. E. (2021). The motor basis for misophonia. *J. Neurosci.* 41 5762–5770. 10.1523/jneurosci.0261-21.2021 34021042 PMC8244967

[B64] Kumar S. Forster H. M. Bailey P. Griffiths T. D. (2008). Mapping unpleasantness of sounds to their auditory representation. *J. Acoust. Soc. Am.* 124 3810–3817. 10.1121/1.3006380 19206807

[B65] Kumar S. Tansley-Hancock O. Sedley W. Winston J. S. Callaghan M. F. Allen M. (2017). The brain basis for misophonia. *Curr. Biol.* 27 527–533. 10.1016/j.cub.2016.12.048 28162895 PMC5321671

[B66] Lakens D. (2013). Calculating and reporting effect sizes to facilitate cumulative science: a practical primer for t-tests and ANOVAs. *Front. Psychol.* 4:863. 10.3389/fpsyg.2013.00863 24324449 PMC3840331

[B67] Lin X. F. Chen Y. Y. Wang M. X. Song C. Lin B. L. Yuan X. P. (2020). Altered topological patterns of gray matter networks in tinnitus: a graph-theoretical-based study. *Front. Neurosci.* 14:541. 10.3389/fnins.2020.00541 32536854 PMC7267018

[B68] McKay D. Acevedo B. P. (2020). Clinical characteristics of misophonia and its relation to sensory processing sensitivity: a critical analysis. *High. Sensitive Brain* Chapter 7 165–185.

[B69] Meeus O. M. Spaepen M. Ridder D. D. Heyning P. H. (2010). Correlation between hyperacusis measurements in daily ENT practice. *Int. J. Audiol.* 49 7–13. 10.3109/14992020903160868 20053152

[B70] Moore B. C. J. Glasberg B. R. (2004). A revised model of loudness perception applied to cochlear hearing loss. *Hear. Res.* 188 70–88. 14759572 10.1016/S0378-5955(03)00347-2

[B71] Moore B. C. J. Glasberg B. R. Baer T. (1997). A model for the prediction of thresholds, loudness, and partial loudness. *J. Audio Eng. Soc.* 45 224–240.

[B72] Naylor J. Caimino C. Scutt P. Hoare D. J. Baguley D. M. (2021). The prevalence and severity of misophonia in a uk undergraduate medical student population and validation of the amsterdam misophonia scale. *Psychiatric Q.* 92 609–619. 10.1007/s11126-020-09825-3 32829440 PMC8110492

[B73] Pigeon W. R. May P. E. Perlis M. L. Ward E. A. Lu N. Talbot N. L. (2009). The effect of interpersonal psychotherapy for depression on insomnia symptoms in a cohort of women with sexual abuse histories. *J. Trauma. Stress* 22 634–638. 10.1002/jts.20456 19885874 PMC2798908

[B74] Porcaro C. K. Alavi E. Gollery T. Danesh A. A. (2019). Misophonia: awareness and responsiveness in academia. *J. Postsecond. Educ. Disabil.* 32 108–118.

[B75] Rosenthal M. Z. Anand D. Cassiello-Robbins C. Williams Z. J. Guetta R. E. Trumbull J. (2021). Development and initial validation of the duke misophonia questionnaire. *Front. Psychol.* 12:709928. 10.3389/fpsyg.2021.709928 34659024 PMC8511674

[B76] Rouw R. Erfanian M. (2018). A large-scale study of misophonia. *J. Clin. Psychol.* 74 453–479. 10.1002/jclp.22500 28561277

[B77] Schecklmann M. Landgrebe M. Langguth B., and Tri Database Study Group (2014). Phenotypic characteristics of hyperacusis in tinnitus. *PLoS One* 9:e86944. 10.1371/journal.pone.0086944 24498000 PMC3908961

[B78] Schroder A. van Diepen R. Mazaheri A. Petropoulos-Petalas D. Soto de Amesti V. Vulink N. (2014). Diminished n1 auditory evoked potentials to oddball stimuli in misophonia patients. *Front. Behav. Neurosci.* 8:123. 10.3389/fnbeh.2014.00123 24782731 PMC3988356

[B79] Schroder A. van Wingen G. Eijsker N. San Giorgi R. Vulink N. C. Turbyne C. (2019). Misophonia is associated with altered brain activity in the auditory cortex and salience network. *Sci. Rep.* 9:7542. 10.1038/s41598-019-44084-8 31101901 PMC6525165

[B80] Schroder A. Vulink N. Denys D. (2013). Misophonia: diagnostic criteria for a new psychiatric disorder. *PLoS One* 8:e54706. 10.1371/journal.pone.0054706 23372758 PMC3553052

[B81] Sheldrake J. Diehl P. U. Schaette R. (2015). Audiometric characteristics of hyperacusis patients. *Front. Neurol.* 6:105. 10.3389/fneur.2015.00105 26029161 PMC4432660

[B82] Sherlock L. P. Formby C. (2005). Estimates of loudness, loudness discomfort, and the auditory dynamic range: normative estimates, comparison of procedures, and test-retest reliability. *J. Am. Acad. Audiol.* 16 85–100. 15807048 10.3766/jaaa.16.2.4

[B83] Siepsiak M. Rosenthal M. Z. Raj-Koziak D. Dragan W. (2022). Psychiatric and audiologic features of misophonia: use of a clinical control group with auditory over-responsivity. *J. Psychosom. Res.* 156:110777. 10.1016/j.jpsychores.2022.110777 35259551

[B84] Siepsiak M. Sliwerski A. Lukasz Dragan W. (2020). Development and psychometric properties of misoquest-a new self-report questionnaire for misophonia. *Int. J. Environ. Res. Public Health* 17:1797. 10.3390/ijerph17051797 32164250 PMC7084437

[B85] Smithson M. (2001). Correct confidence intervals for various regression effect sizes and parameters: The importance of noncentral distributions in computing intervals. *Educ. Psychol. Meas.* 61 605–632.

[B86] StataCorp (2013). *Stata Statistical Software: Release 13.* College Station, TX: StataCorp.

[B87] Swedo S. Baguley D. M. Denys D. Dixon L. J. Erfanian M. Fioretti A. (2022). A consensus definition of misophonia: using a delphi process to reach expert agreement. *Front. Neurosci.* 16:841816. 10.3389/fnins.2022.841816 35368272 PMC8969743

[B88] Sztuka A. Pospiech L. Gawron W. Dudek K. (2010). DPOAE in estimation of the function of the cochlea in tinnitus patients with normal hearing. *Auris Nasus Larynx* 37 55–60. 10.1016/j.anl.2009.05.001 19560298

[B89] The MathWorks (2020). *MATLAB and Statistics Toolbox.* Natick, MA: The MathWorks, Inc.

[B90] Tyler R. S. Pienkowski M. Rojas Roncancio E. Jun H. J. Brozoski T. Dauman N. (2014). A review of hyperacusis and future directions: part I. Definitions and manifestations. *Am. J. Audiol.* 23 402–419. 10.1044/2014_aja-14-0010 25104073

[B91] Wu M. S. Lewin A. B. Murphy T. K. Storch E. A. (2014). Misophonia: incidence, phenomenology, and clinical correlates in an undergraduate student sample. *J. Clin. Psychol.* 70 994–1007. 10.1002/jclp.22098 24752915

[B92] Zaugg T. L. Thielman E. J. Griest S. Henry J. A. (2016). Subjective reports of trouble tolerating sound in daily life versus loudness discomfort levels. *Am. J. Audiol.* 25 359–363. 10.1044/2016_aja-15-0034 27768802

[B93] Zhou X. Wu M. S. Storch E. A. (2017). Misophonia symptoms among Chinese university students: Incidence, associated impairment, and clinical correlates. *J. Obsessive Compuls. Relat. Disord.* 14 7–12.

